# Human Adenovirus Species D Interactions with Corneal Stromal Cells

**DOI:** 10.3390/v13122505

**Published:** 2021-12-14

**Authors:** Jaya Rajaiya, Amrita Saha, Xiaohong Zhou, James Chodosh

**Affiliations:** Massachusetts Eye and Ear, Harvard Medical School, Boston, MA 02114, USA; Amrita_Saha@meei.harvard.edu (A.S.); Xiaohong_Zhou@meei.harvard.edu (X.Z.)

**Keywords:** adenovirus, human adenovirus species D, epidemic keratoconjunctivitis, adenovirus keratitis, intracellular signaling, keratocyte

## Abstract

Notable among the many communicable agents known to infect the human cornea is the human adenovirus, with less than ten adenoviruses having corneal tropism out of more than 100 known types. The syndrome of epidemic keratoconjunctivitis (EKC), caused principally by human adenovirus, presents acutely with epithelial keratitis, and later with stromal keratitis that can be chronic and recurrent. In this review, we discuss the current state of knowledge regarding the molecular biology of adenovirus infection of corneal stromal cells, among which the fibroblast-like keratocyte is the most predominant, in order to elucidate basic pathophysiologic mechanisms of stromal keratitis in the human patient with EKC.

## 1. Introduction

The cornea is the transparent window of the eye. The cornea is also a mucosal surface and frequently encounters external infectious agents, whether airborne, through hand-to-eye contact, on fomites or contact lenses, or due to trauma. Corneal infection can lead to scarring, reduced vision, and when particularly severe, perforation and loss of the eye. An important and often overlooked cause of corneal infection is the highly transmissible adenovirus, more often recognized as the most common cause of infectious conjunctivitis [1]. The conjunctiva is the mucous membrane that abuts the cornea, surfaces the rest of the external globe, and lines the internal surface of the eyelids. Infections of the conjunctiva are medically important; about 6 million infectious conjunctivitis cases present to clinicians annually in the United States, at an estimated cost of hundreds of millions of US dollars for diagnosis and treatment annually [2]. Over half of all conjunctivitis cases are caused by adenovirus [1,3,4], and in an undetermined but significant proportion of these cases, the cornea is also infected. In such cases, the presentation is referred to as epidemic keratoconjunctivitis, or EKC [5]. EKC is highly contagious, outbreaks occur world-wide [6,7,8,9,10,11,12,13,14,15,16,17,18,19], and to date, there is no effective therapy to safely mitigate morbidity from the disorder [20].

The adenovirus is non-enveloped, icosahedral shaped, and contains a double stranded DNA genome of ≈36,000 base pairs. The virion capsid contains 240 hexon and 12 penton capsomers, and assorted minor capsid proteins. Each penton capsomer forms the base (hence, “penton base”) for a trimeric fiber protein, the principal ligand for the various host cell receptors [21,22,23,24,25,26,27]. Receptor binding leads to secondary interaction between an RGD motif in each penton base protein with cellular integrins [28,29,30], e.g., α_v_β_3_, that then aggregate, phosphorylate, and activate Src [31,32], to induce endocytosis of the virus. Human adenoviruses (HAdV) divide phylogenetically into 7 species (A-G), with now over 100 types [33,34]. HAdV infections are an important source of morbidity and mortality world-wide, through readily transmittable infections at mucosal sites [35]. Infection may be especially lethal in infants [36,37,38,39] and the immune compromised [40,41,42,43], but can also lead to fatal acute respiratory distress syndrome in healthy adults [44,45]. HAdV infections of the eye present as either simple follicular conjunctivitis, pharyngoconjunctival fever, or EKC. The first two are self-limited and do not disturb the cornea. In contrast, in EKC, the cornea is directly infected. The major EKC pathogens fall within species D: types 8, 37, 53, 54, 56, 64 (previously 19a), and 85 (recently emerged) [20,46,47,48,49,50,51,52,53,54]. In a recent comprehensive phylogenomics study of archived adenovirus sequences, the members of HAdV-D were uniquely distinct from all other adenoviruses found in human and non-human primates [55].

## 2. Cornea Organogenesis, Structure, and Response to Injury/Infection

**Figure 1:**
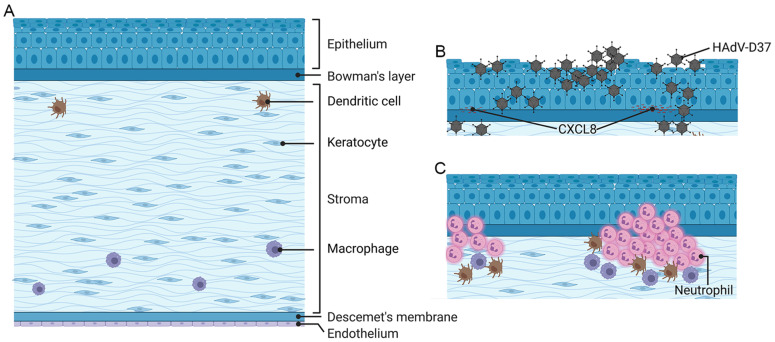
Schematic of the human cornea in cross-section. In the normal, uninfected eye (**A**), the central corneal epithelium is intact, and the corneal stroma is sparsely populated by fibroblast-like keratocytes and a smaller number of myeloid cells. In the cornea acutely infected by a cornea-tropic adenovirus (**B**), the corneal epithelium is disrupted by virus-induced cytopathic effect. Upon infection of superficial keratocytes, chemokines, e.g., CXCL8, accumulate in multifocal loci at the level of the corneal epithelial basement membrane. In a subset of infected eyes, after the epithelium has healed (**C**), neutrophils and other monocytes migrate from corneal limbal blood vessels to form multifocal, subepithelial infiltrates at foci of chemokine accumulation. Created with BioRender.com under a standard academic license.

The anterior eye tissue is derived from the surface ectoderm, mesoderm, and neural crest, while the posterior eye tissue develops from the neural tube ectoderm [56]. The cornea’s function is to focus light onto the (posterior) neural retina; visual information is then sent to the brain via the optic nerve. The cornea performs ≈80% of light bending/focusing for vision; the remainder is performed by the crystalline lens, which also serves to adjust the eye’s focus from far to near (accommodation). The cornea is the most anterior eye tissue and, from front to back (Figure 1A), is composed of corneal epithelium, epithelial basement membrane, Bowman’s layer, stroma, Descemet’s membrane, and the single-cell layer-thick corneal endothelium. Descemet’s membrane is the basement membrane of the corneal endothelium. The corneal epithelium measures about 50 µm in thickness and is composed of four to six layers of nonkeratinized, stratified squamous cells. There is constant repopulation of epithelial cells through differentiation and maturation of transient amplifying cells at the basal layer, which in turn have migrated through the deep corneal epithelial layer from the peripheral corneal “limbus”, the site of corneal epithelial stem cells [57]. Superficial epithelial cells have a life span of 7–10 days, after which they undergo apoptosis and desquamation [58]. The corneal epithelium serves as a relative barrier to pathogens and other foreign particles through surface mucins and intercellular tight junctions, protecting the underlying corneal stroma. The corneal epithelium is the most highly innervated mucosal surface with ≈7000 nociceptors per square mm [59], rendering it more sensitive than skin by several orders of magnitude [60]. The corneal epithelium, like other epithelial surfaces, also expresses membrane-associated mucins (MAMs), and viruses must first negotiate a MAM-rich glycocalyx in order to infect corneal epithelial cells. One specific MAM, MUC16, has been shown to be particularly important in ocular surface defense [61]. MUC16 expressed by corneal epithelial cells has been shown to directly impact adenovirus tropism for the eye. Menon and coworkers demonstrated that the EKC-causing human adenovirus species D type 37 (HAdV-D37), but not the highly similar but non-EKC-associated HAdV-D19, induced ectodomain release of corneal epithelial MUC16, reducing barrier function to infection [62]. Corneal epithelial cells also express Toll-like receptor (TLR) 2 and TLR4, but their expression under normal conditions is intracellular, i.e., not on the cell surface [63]. Therefore, corneal epithelial TLRs are not directly activated by commensal bacteria on the ocular surface, thus rendering a relatively “immune-silent” environment. However, adenovirus infection of the conjunctiva induces significant inflammation, and the associated conjunctival discharge, replete with proinflammatory cytokines [64,65], very likely alters corneal epithelial cell TLR expression. Dendritic cells (DCs) are also present in normal human corneal epithelium, mostly in the corneal periphery. CD11c^+^CD16^−^ DCs are the most predominant, with smaller numbers of CD11c^+^CD16^+^ and CD11c^+^CD1c^+^ cells [66,67]. Innate immune responses upon adenovirus infection of dendritic cells at other mucosal sites have been well studied [68,69,70,71,72,73].

Directly below the corneal epithelium is the epithelial basement membrane, laid down by basal epithelial cells. The corneal epithelial basement membrane is rich in heparan sulfate proteoglycans which promote epithelial cell migration, proliferation, and differentiation [74]. Corneal epithelial basement membrane very effectively binds positively charged chemokines, thus establishing relatively stable reservoirs that may serve as initiation sites for recurrent accumulations of leukocytes, characteristic of post-EKC keratitis, and manifest clinically by multifocal, corneal subepithelial infiltrates (SEI) [75]. The corneal stroma sits just beneath the epithelial basement membrane. The most superficial corneal stroma in humans, known as Bowman’s layer, is distinct histologically as an acellular layer of 15–18 µm in thickness, consisting mostly of collagen type I. Its function is unknown, but it is permeable to macromolecules [76]. Below the Bowman’s layer is a sparsely cellular stroma that contributes to the majority of the corneal thickness (≈90%), approximately 550 µm centrally but closer to 1 mm in the cornea periphery. A highly organized network of lamellar collagen fibers in the stroma confers transparency and consists largely of heterodimeric complexes of collagen types I and V [77]. Other proteins in the stroma include lumican and keratocan, the major keratan sulfate proteoglycans. The principal cell type in the stroma (constituting ≈94% of corneal stromal cells) is the neural crest derived keratocyte. These cells produce collagen and glycosaminoglycans, and maintain corneal transparency through modulation of corneal stromal extracellular matrix [78,79,80] and expression of intracellular crystallins [81]. Keratocytes are relatively quiescent and rarely undergo cell division, but in injury or infection, they are transformed to fibroblasts and myofibroblasts [82]. Although keratocytes make up the great majority of cells in the corneal stroma, ≈6% of the total cell population in the corneal stroma are CD45^+^ cells of monocytic lineage [83]. CD45^+^ CD11c^+^ cells are found mostly within the anterior stroma, and CD11c^−^ CD11b^+^ cells within the posterior stroma [67]. In a study utilizing the mouse model of adenovirus keratitis [84], macrophage Fas-Induced apoptosis (MaFIA) transgenic mice, in which the mouse colony stimulating factor 1 receptor promoter (*Csf1r*) was used to drive expression of a mutant human FK506 binding protein 1A in macrophages and dendritic cells to induce apoptosis, clinical keratitis was reduced and the recruitment of leukocytes was diminished as compared to controls [85]. In addition to monocytes in the cornea, very small numbers of plasmacytoid DCs are also present in the corneal stroma, as first shown by Sosnova and co-authors [86]. A role for these cells in adenovirus keratitis has not been explored. The very posterior cornea is bound by Descemet’s membrane and the corneal endothelium.

Corneal epithelial and stromal cells communicate together to regulate and maintain corneal homeostasis [87,88,89]. Such crosstalk is critical to the corneal response to injury [90]. Upon disruption of the epithelial barrier, whether by injury or infection, even when isolated to the overlying corneal epithelium alone, those keratocytes that do not take on the phenotype of fibroblasts or myofibroblasts die due to apoptosis [91,92,93,94]. They are repopulated upon injury by a subpopulation of corneal stromal stem cells [95]. Myofibroblasts have reduced crystalline expression and therefore lack transparency and directly contribute to corneal opacity [82,96,97,98]. Keratocytes which have been activated to the fibroblast phenotype express TLR1-TLR7, TLR9, and TLR10 [99,100,101,102,103,104]. Both fibroblasts and myofibroblasts participate in the response to injury and in tissue remodeling. This is similar to the role of fibroblasts elsewhere, for example in the heart, in which crosstalk between fibroblasts and cardiomyocytes is critical to both cardiac development and repair after tissue injury [105,106]. Corneal fibroblasts ably detect and respond to pathogen-associated molecular patterns (PAMPs) from microbes through the expression of TLRs. They express cytokines, chemokines, and adhesion molecules that are responsible for the recruitment of inflammatory cells [102,107,108]. Our past studies of adenovirus infection of human corneal fibroblasts in vitro and in a mouse model of adenovirus keratitis in vivo showed early expression of CXCL8 and its murine homologue CXCL1, respectively; and CCL2, and ICAM-1, all within the first day post infection (pi) [32,75,84,109]. In addition to a role in the acute keratitis of EKC, and based on additional and compelling evidence for a critical role of tissue fibroblasts in the maintenance of chronic inflammation [110,111,112,113], we speculate that human corneal fibroblasts also play a role in the pathogenesis of the chronic, recurrent stromal keratitis that often follows EKC [5].

## 3. Epidemic Keratoconjunctivitis (EKC)

EKC is characterized clinically by follicular lymphoid hyperplasia of the conjunctiva, preauricular lymphadenopathy, and punctate or geographic epithelial keratitis (Figure 1B), with an explosive clinical course [114,115]. The contralateral eye is affected in ≈70% [116]. Inflammatory conjunctival membranes form in ¼–½ of infected eyes [5,115]; if untreated, they become incorporated into the host tissue and can form adhesions (symblephara) that may restrict ocular motility [117]. In EKC, rapid onset and severity are distinctive, but the hallmark is corneal involvement [115,118,119]. The epithelial keratitis induced by viral cytopathic effect resolves within days, but stromal keratitis in the form of SEI then ensues (in 60% of cases in a recent large study) [52], typically appearing at 14–21 days pi.

SEI can be recalcitrant to treatment. While full resolution can occur within a few weeks after onset, in a significant proportion of patients, the keratitis will persist or recur for months to years pi (in one study, 47% at 2 years pi) [5,120,121], causing scarring [122], irregular astigmatism, glare, foreign body sensation, and blurred vision [123]. Rajaiya and coworkers [75] studied the ontogeny of adenovirus-induced, corneal SEI, by modifying a previously published 3D human corneal “facsimile” of adenovirus keratitis [124]. The original facsimile was composed of human keratocytes and type I collagen plated on transwell plates; Rajaiya added an overlying layer of Matrigel^®^ to the model to simulate an epithelial basement membrane. The facsimiles were plated on transwell plates with a 3 μm pore size and infected overnight with HAdV-D37. When freshly isolated human peripheral blood leukocytes were placed beneath the inserts, within 1 h, neutrophils had migrated upward (against gravity) to form focal infiltrates that mimicked SEI and co-localized with CXCL8 bound to heparan sulfate in the Matrigel.

HAdVs do not replicate in mouse cells [125,126,127,128]. However, a novel mouse model of adenovirus keratitis [46,84,129,130,131] has permitted study of the innate immune responses to the virus. Injection with ≥5 × 10^4^ tissue culture infectious doses (TCID) of the virulent EKC pathogen HAdV-D37 [50,132,133,134,135], directly into the mouse corneal stroma by a heat-pulled glass micropipette needle, bypasses the nonpermissive mouse ocular surface and induces a stromal keratitis that peaks ≈4 days pi [136], and then resolves, only to recur later in a subset of infected mice (Figure 1C) [84]. HAdV-D37 empty capsid (no viral DNA), is sufficient to induce clinically evident keratitis; viral replication is unnecessary (and does not occur) [130]. Neutrophil chemotaxis into the adenovirus infected cornea is dependent on CXCL1 and its receptor, CXCR2 [129]. Leukocytic infiltrates and CXCL1 expression in the mouse can be blocked by treatment with a monomer containing RGD but not by a control peptide, consistent with capsid driven inflammation [130]. Subsequent investigations into TLR activation in the mouse adenovirus keratitis model showed Src kinase associated activation of MyD88 [131]. Keratitis was reduced in MyD88 knockout mice, as well as in mice knocked out for both TLR2 and TLR9.

As viruses within HAdV-D do not replicate in murine cells, detailed in vitro studies of the cellular response to adenovirus infection of the cornea have relied extensively on human keratocytes cultured from deceased human corneal donors. These studies were initiated after publication of a seminal study showing that adenovirus type 5 (HAdV-C5) infection of HeLa cells induces interleukin-8 (CXCL8) expression through a Raf/mitogen-activated protein (MAP) kinase signaling pathway [137], and based on reasoning that CXCL8 expression by infected corneal cells might be responsible for the keratitis seen in EKC. The earliest studies utilized a virus isolated from the cornea of a patient with acute EKC that was initially characterized as HAdV-D19c [118]. This virus was later whole genome sequenced [51], and found to contain only the hexon hypervariable regions from type 19 (≈3% of the total genome), with the majority of the genome recombinant with HAdV-D37 [49], a highly virulent cause of EKC. HAdV-D19c was then classified as a novel type under criteria accepted by GenBank [138], and renamed as HAdV-D64 [49]. Primary cultures of keratocytes were chosen for the infection model, after it was found that corneal epithelial cells, although susceptible to adenovirus infection, expressed cytokines in very limited amounts in comparison to log-unit increases in expression by adenovirus-infected keratocytes [32].

**Figure 2:**
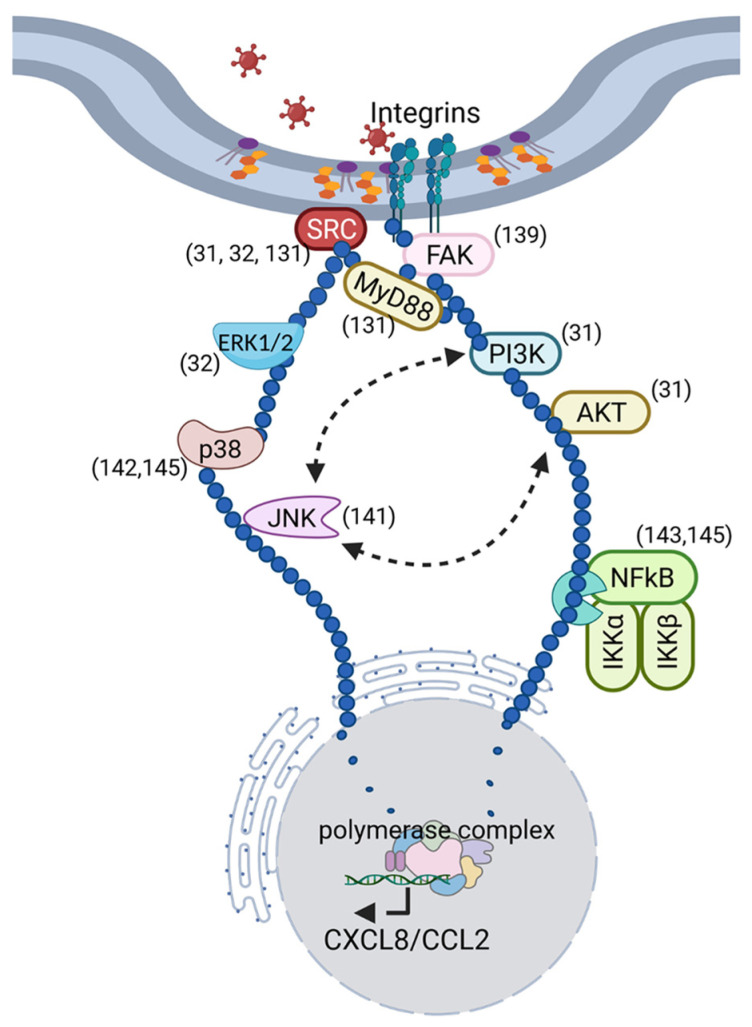
Schematic summarizes the known intracellular signaling events in human keratocytes upon adenovirus infection. Activation of intracellular signaling is initiated upon integrin binding and viral entry, prior to the earliest adenoviral gene expression. Signaling leads to chemokine expression with subsequent formation of subepithelial leukocyte infiltrates at chemokine sites of binding to epithelial basement membrane, and to maintenance of cell viability to enable viral replication. The dark blue circles reflect the relationships between intracellular signaling molecules that occur upon adenovirus infection, culminating in nuclear translocation of the transcription factors shown, and leading to host proinflammatory gene expression. Citations for each signaling molecule activated by adenovirus infection are shown in parentheses. Created with BioRender.com under a standard academic license.

As discussed above, primary keratocytes when cultured in the presence of serum take on the morphology of fibroblasts. Early studies using HAdV-D64 identified several tyrosine kinases as critical to the expression of CXCL8 (Figure 2). For example, cytosolic focal adhesion kinase (FAK) was shown to be activated (phosphorylated) within 15 min of infection, well before the onset of adenoviral gene expression, and its chemical inhibition reduced CXCL8 expression [139]. FAK activation is known to induce changes to the cytoskeleton [140], consistent with a role in the altered cellular morphology previously shown to occur in adenovirus infected cells [28]. In keratocytes infected with HAdV-D64, phosphorylated FAK accumulated in relatively greater abundance in the Triton X-insoluble cell pellet [139]. Further studies using ultraviolet (UV) light-inactivated virus, which enters cells like an unaltered wild-type virus but does not replicate, still induced CXCL8 expression [32]. Heat-inactivated viruses, which do not enter cells due to heat-induced damage to protein ligands on the external capsid surface, did not induce CXCL8. Furthermore, the general tyrosine kinase inhibitor, herbimycin, blocked CXCL8 expression in infected cells. Src was the first tyrosine kinase induced; phosphorylation was observed within 5 min of infection. Phosphorylation of the extracellular signal-regulated kinases (ERK) 1/2 was seen within 15 min of infection. Chemical inhibitors of Src and ERK1/2 blocked CXCL8 expression. It was subsequently shown that adenovirus infection of keratocytes also activated phosphoinositide 3-kinase (PI3K) and downstream protein kinase B (AKT) [31], in a pathway that appeared to also involve Src. Activation of the PI3K/AKT pathway was found to protect infected keratocytes from apoptotic cell death, whereas chemical or siRNA knock down led to rapid apoptotic cell death upon infection. Notably, early viral gene expression after infection occurred despite AKT knock down, but cells died prior to viral replication, suggesting that induction of this pathway by adenovirus acts to sustain cell viability and enable viral replication. Therefore, blockade of the PI3K/AKT pathway may represent a viable target for anti-adenoviral therapy.

In vitro studies showing activation of ERK1/2 downstream of Src led to investigation of other MAP kinases, including JNK [141] and p38 [142]; both were phosphorylated within 15 to 30 min pi. While ERK1/2 and p38 were each shown to activate expression of CXCL8, activation of JNK induced expression of CCL2 (monocyte chemoattractant protein-1, MCP-1) but not CXCL8, through activation of the transcription factor c-Jun. All three MAP kinases were induced by adenovirus infection through upstream activation of Src, and inhibitors of each pathway differentially inhibited translocation of different NFkappaB (NFκB) subunits [143]. Differential activation of NFκB was found to control the specific pattern of chemokine expression and was time dependent. Transactivation of the CXCL8 promotor occurred within one hour, upon binding of NFκB p65 and p50 subunits, while NFκB cREL binding to the CCL2 promotor occurred at ≈4 h pi. PP2, an inhibitor of Src, fully blocked NFκB translocation. These findings correlated well with those from the mouse model of adenovirus keratitis, in which CXCL1 expression and resultant neutrophil infiltration occurred rapidly, within 1 day pi, while CCL2 expression and monocyte infiltration were delayed [84]. Notably, injection of PP2 into the mouse cornea prior to infection reduced clinical signs of adenovirus keratitis and CXCL1 expression, as compared to controls [144]. PP2 also blocked CXCL8 expression and neutrophil infiltrates in the human corneal facsimile model of adenovirus keratitis [75].

Later studies showed a role for heat shock protein (HSP) 27 in infection by HAdV-D37 in a signalosome that included p38 and NFκB p65 [145]; siRNA knockdown of HSP27 reduced CXCL8 expression. Studies further upstream indicated that viral entry into keratocytes was dependent on cell membrane lipid rafts and caveolin-1 within minutes of infection [144]. HAdV-D37 DNA was found in caveolin-1 containing endosomal fractions and by immunoelectron microscopy; caveolin-1 and HAdV-37 virions were colocalized to intracellular vesicles. Importantly, Src phosphorylation and CXCL1 expression were both reduced in HAdV-D37 infection of corneas in caveolin-1 knockout mice, relative to wild-type mice. In contrast, entry of HAdV-D37 into human corneal epithelial cells was found to occur by a noncanonical clathrin-mediated pathway not involving caveolin-1 [146].

The cornea quite literally forms the window of the eye. The uniqueness of its design stems from its function as the major site of light bending and transparency for focusing of light on the retina. The cornea also serves as a barrier between the external world and the highly vulnerable internal eye. Its highly specific cellular and extracellular organization renders it uniquely susceptible to damage by infectious organisms that might otherwise be less impactful. For example, infections of the conjunctiva by the very same viruses that infect the cornea typically resolve without significant long-term sequelae. However, much remains unknown about adenovirus infection of the cornea, including, for example, the interactions between the virus and resident monocytes, and the potential interactions between keratocytes and resident monocytes in the initial response to infection. Also unknown is why subepithelial infiltrates can recur months to years after the acute infection. Future studies are needed to elucidate cellular interactions during acute infection, and how acute infection alters the cornea for the long term.
